# The rarest invaders: systematic global evidence for the conservation-invasion paradox in plants

**DOI:** 10.1038/s44185-026-00142-9

**Published:** 2026-06-19

**Authors:** Ramiro R. Ripa, Yves P. Klinger, Jorgelina Franzese

**Affiliations:** 1 Division of Landscape Ecology and Landscape Planning, Justus Liebig University Gießen, Gießen, Germany; 2 Investigaciones de Ecología en Ambientes Antropizados, Instituto de Investigaciones en Biodiversidad y Medioambiente (Universidad Nacional del Comahue - CONICET), S. C. Bariloche, Río Negro Argentina

**Keywords:** Ecology, Ecology, Plant sciences

## Abstract

The Conservation–Invasion Paradox (CIP) refers to species that are threatened or declining within their native ranges establish invasive populations elsewhere. While this paradox has been documented primarily in animals, its prevalence, structure, and drivers in plants remain poorly understood. Investigating the CIP is crucial for linking mechanisms that underlie both species decline and invasion success, and offers insights into biodiversity change in the Anthropocene. Here, we provide the first global, systematic assessment of the CIP in vascular plants by integrating three global alien flora databases with three conservation assessment databases. Using reproducible cross-database criteria (the simultaneous occurrence of invasive status in at least one global alien flora database and conservation concern in at least one global conservation assessment database), we identified 89 plant species under the CIP. Threatened plant species were approximately 10 times less likely to naturalize and 17 times less likely to become invasive than non-threatened species, making the CIP a genuinely paradoxical outcome. CIP plant species are concentrated in the Americas and East-Southeast Asia and exhibit strong anthropogenic signals, with dominant human uses and persistent threats from agriculture, overexploitation, and development. Our findings demonstrate that the CIP arises from a spatial decoupling between invasion success and native range persistence, driven by the same human valuation processes that promotes translocation and intensify native-range pressures. The CIP also exposes a mismatch between biological invasion and conservation frameworks, where separate regulatory systems independently assess invasive status and conservation concern, revealing a governance gap for species exhibiting both conditions simultaneously.

## Introduction

Invasive non-native plant species (INNP) have traditionally been framed as ecological “super competitors”, characterized by high fecundity, rapid growth, broad environmental tolerance, and superior resource acquisition, traits commonly invoked to explain their dominance in novel ecosystems^1–3^. However, this trait-centered perspective has increasingly been challenged by evidence showing that invasion success is neither universal nor consistently predicted by intrinsic ecological characteristics alone. Comparative syntheses and global analyses indicate that many traits associated with invasion success are tightly related to human-mediated introduction histories, repeated translocation events, and propagule pressure, rather than reflecting inherent ecological superiority^4–6^. At broad spatial scales, the probability of establishment and naturalization of non-native plants is more strongly associated with the intensity and frequency of human introductions, patterns of use, and socio-economic selection, propagule pressure, and colonization pressure than with functional traits in isolation^2,7–10^.

**Fig. 1:**
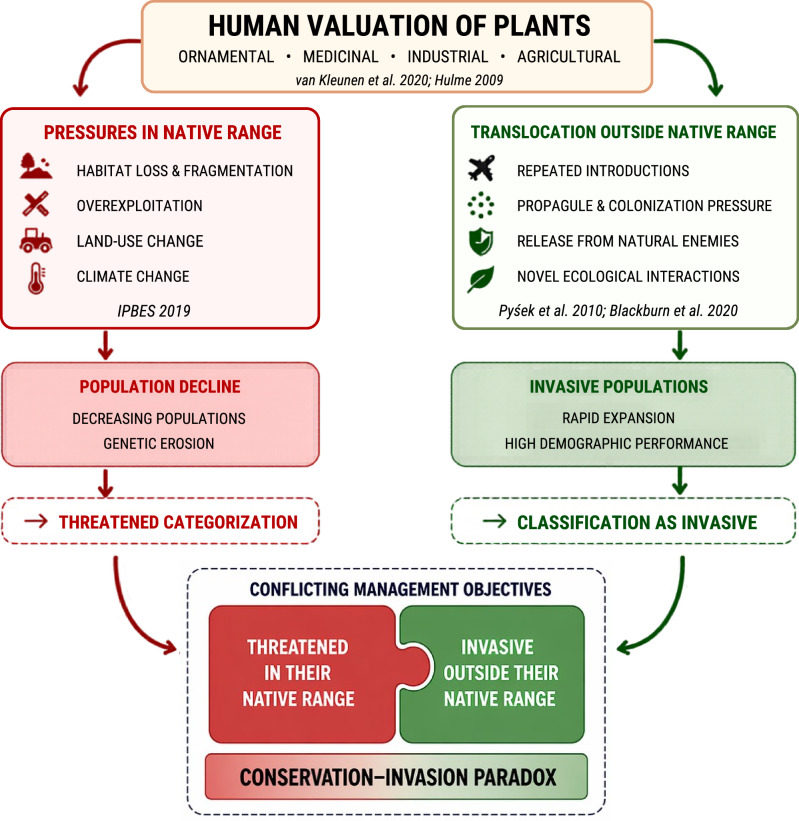
Conceptual framework of the conservation-invasion paradox (CIP) in plants. Human valuation of plant species, expressed through different uses, simultaneously structures the processes operating in native and non-native ranges. In the native range, human pressures can lead to population decline, genetic erosion, and the threat of extinction. Outside the native range, deliberate or unintentional translocation associated with human activities gives rise to invasion processes. The spatial disconnection of these processes generates the conservation–invasion paradox, whereby the same species is invasive in its introduced range while declining or threatened in its native range.

In this context, plants valued for ornamental, medicinal, industrial, or agricultural purposes are disproportionately represented among species translocated beyond their native ranges, reflecting long-standing links between social valuation, intentional translocation and persistence in human-modified landscapes^5,11^ (Fig. 1). Crucially, this same human valuation can generate contrasting demographic outcomes across ranges: while facilitating spread and population expansion outside the native range, it may co-occur with sustained anthropogenic pressures within the native range, including overexploitation, habitat loss, and land-use change (Fig. 1). This dual and spatially decoupled influence of human valuation can lead to the Conservation–Invasion Paradox (CIP;^12^, in which species become successful invaders beyond their native range while simultaneously declining or becoming threatened in their areas of origin (Fig. 1). Although some studies have recently addressed the CIP, most research has focused on animal species^12–15^, leaving INNP underrepresented. Recent work on naturalized plants has nonetheless revealed that threatened species can be disproportionately represented among established introduced floras, suggesting that coinciding invasion success and native-range decline can also occur in plants^16^.

Across taxa in which the CIP has previously been examined, the phenomenon does not appear to arise randomly, but is instead associated with recurrent patterns^12,13,17^. CIP animal species are frequently characterized by deliberate introductions, commercial exploitation, and, in some cases, active human management that facilitates population establishment and expansion beyond their native ranges^12,13,17–19^. At the same time, these species continue to experience active and often intensifying pressures within their native ranges, such as habitat loss, overexploitation, and landscape transformation, indicating that invasion success elsewhere does not alleviate native-range vulnerability^12,13^. These studies also suggest that rather than being evenly distributed worldwide, the CIP exhibits marked geographical asymmetries, with species concentrated in Asia and Australia, reflecting uneven histories of human use, environmental pressure, and biogeographic vulnerability^13,15^.

The CIP poses significant challenges for biodiversity conservation and ecosystem management in the context of accelerating global change. Species redistribution, anthropogenic disturbances, and conflicts between conservation and control objectives complicate the application of conventional management approaches. A disconnect between invasion biology and conservation policy can contribute to mismatches in management priorities, which may be especially pronounced in species exhibiting such contrasting biogeographical dynamics^20,21^. Recent studies^12,14^ show that, in some CIP contexts, introduced populations may acquire conservation relevance when native populations continue to decline or face severe demographic or genetic constraints, generating a conundrum between eradication objectives and species conservation at a global scale. From a broader perspective, the value of maintaining populations outside their original context is well established within ex situ conservation frameworks^22^, where such populations can function as demographic or genetic reservoirs, insurance against extinction, or even sources for the restoration of native populations^22^. While invasive populations are not equivalent to formally managed ex situ collections, their existence in CIP contexts challenges management responses based exclusively on invasion status^12,13^. A critical step toward understanding and addressing the CIP is the systematic identification of plant species that simultaneously exhibit invasion success outside their native ranges and conservation concern within them.

This study investigates the global occurrence of vascular plant species that are invasive beyond their native distributions while being classified as threatened in the wild in their native ranges. By integrating three global non-native flora databases (Laginhas & Bradley^23^; GRIIS^24^; GloNAF 2.0^25^) with three conservation assessment databases (IUCN Red List v2024-2^26^; BGCI ThreatSearch^27^; NatureServe^28^), we provide the first extensive, statistically grounded assessment of the CIP in vascular plants. Specifically, we ask: (i) whether threatened plant species differ from non-threatened species in their likelihood of becoming naturalized or invasive, thereby assessing whether the CIP represents a genuinely paradoxical pattern in plants; (ii) how many plant species simultaneously meet invasive and conservation concern criteria across multiple global databases; and (iii) whether consistent biological, geographic, and anthropogenic patterns underlie the CIP in plants. By providing the first global assessment of CIP in vascular plants, this study aims to clarify whether this paradox reflects isolated cases or a feature of the Anthropocene, and to identify the contexts in which invasion success and native range vulnerability are closely related.

## Results

### Invasion rates and conservation status of threatened plants

**Fig. 2:**
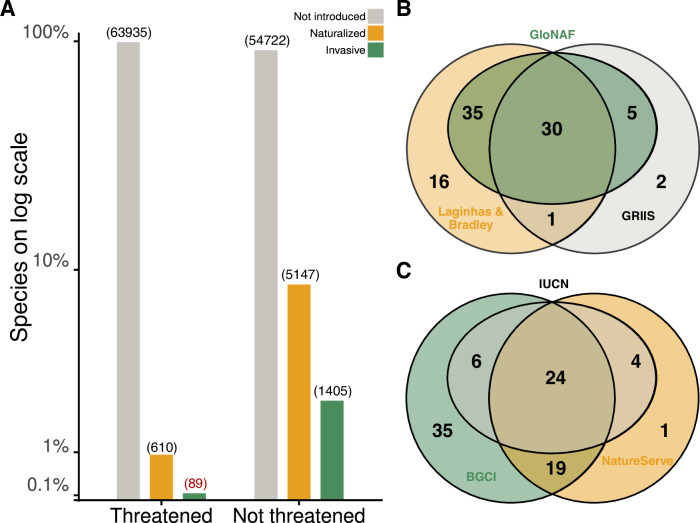
Naturalization and invasion rates of threatened vs. non-threatened plants, and species overlap across databases. **A** Proportion of threatened (*n* = 64,545) and non-threatened (*n* = 59,869) plant species recorded as naturalized or invasive in global non-native flora databases, shown on a logarithmic scale. Species with no introduced records are shown for reference (gray bars). Odds ratios (OR) compare naturalization and invasion rates between threatened and non-threatened species (both *p* < 0.001). **B** Overlap among invasion databases for the 89 CIP species (invasive + threatened): Laginhas & Bradley, GRIIS, and GloNAF 2.0. **C** Overlap among conservation databases for the same 89 CIP species: IUCN Red List, NatureServe, and BGCI ThreatSearch.

Overall, we identified 610 plant species simultaneously classified as introduced and under conservation concern across 124,414 species with documented conservation status. Of these, 460 were naturalized, 61 introduced, and 89 invasive outside their native range (Supplementary Table 2), the latter thereby meeting the criteria for the CIP. Threatened plant species were approximately 10 times less likely to be naturalized (OR = 0.101, 95% CI: 0.093–0.110, *p* < 0.001) and 17 times less likely to be invasive (OR = 0.057, 95% CI: 0.046–0.071, *p* < 0.001) than non-threatened species (Fig. 2A). Among the 89 CIP species, GloNAF 2.0 provided the broadest invasion coverage (70 species) but contributed no exclusively detected species, as all its records overlapped with at least one other database; Laginhas & Bradley covered 82 species and contributed the largest number of unique records (16 species detected exclusively by this source); GRIIS covered 38 species and added only two unique records, with the remaining 36 co-occurring with Laginhas & Bradley and/or GloNAF (Fig. 2B). Thirty species were simultaneously recorded across all three invasion databases. For conservation status, BGCI ThreatSearch contained the most (84) species and contributed the largest number of exclusively detected species (35), IUCN covered 48 species but added only one exclusive record, with the remaining 47 overlapping with BGCI and/or NatureServe; NatureServe covered 34 species and contributed no exclusively detected records (Fig. 2C). Twenty-four species were simultaneously assessed across all three conservation databases.

**Fig. 3:**
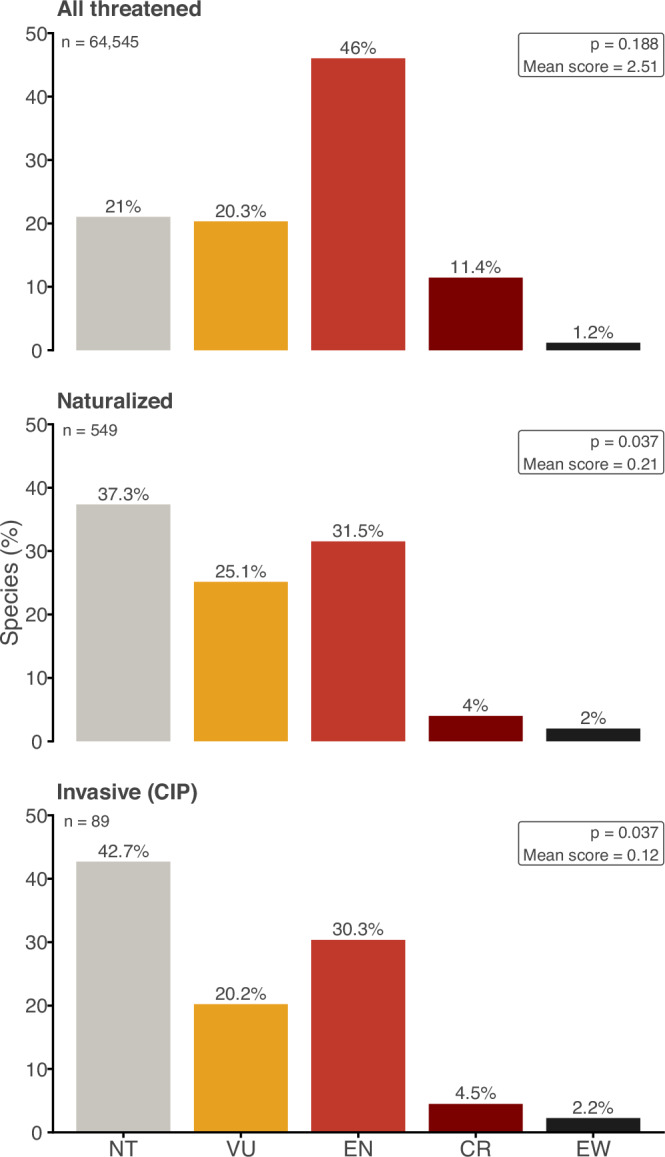
Conservation status distribution across invasion stages for threatened and introduced plants. Proportion of species (%) in each conservation status category (Near Threatened [NT], Vulnerable [VU], Endangered [EN], Critically Endangered [CR], and Extinct in the Wild [EW]) across four groups: all threatened species (*n* = 124,414), species with any record as non-native (*n* = 5757), naturalized species (*n* = 5457), and CIP species (Invasive; *n* = 1494). Mean threat scores, calculated from an ordinal scale based on threat severity, are shown for each group.

A consistent shift in conservation status distribution was observed across the stages of the invasion process (Fig. 3). Species that progressed further along the invasion were classified in less severe threat categories, whereas representation in higher-risk categories declined. This is reflected in a progressive mean threat score decrease from the full set of threatened species (mean = 2.51) to naturalized species (0.21) and invasive species (0.12). Accordingly, species that successfully became invasive were, on average, less threatened than those that only naturalized, and substantially less threatened than the overall pool of species under conservation concern. This directional shift was statistically significant for both non-native subsets (*p* = 0.037). Within the subset of CIP species, conservation status remained heterogeneous but strongly skewed toward lower-risk categories (Fig. 3). These species were distributed across the five threat classes, with the highest representation in Near Threatened (42.7%), followed by Endangered (EN) (30.3%) and Vulnerable (20.2%), whereas Critically Endangered (CE, 4.5%) and Extinct in the Wild (EW) (2.2%) were comparatively rare (Fig. 2D). This distribution contrasts with the full set of species under conservation concern, where higher-risk categories are more prominent (Fig. 3). Only four of the 89 CIP species (4.5%) were listed in CITES appendices, all under Appendix II: *Cedrela odorata* (Neotropical populations), *Pterocarpus indicus*, *Pterocarpus macrocarpus*, and *Vanilla planifolia*.

### Geographic structure, uses, threats, and ecological impacts

**Fig. 4:**
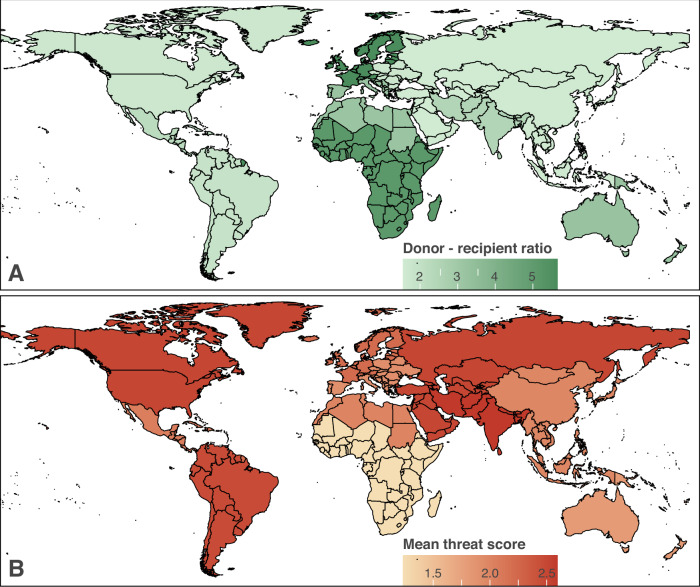
Geographic patterns of plant species exhibiting the Conservation–Invasion Paradox (CIP). **A** Regional distribution of CIP species expressed as the ratio of non-native to native occurrences, indicating the relative imbalance between recipient and donor roles across regions (higher values denote stronger recipient bias). **B** Mean conservation threat score of native CIP species across regions, derived from standardized conservation categories, where higher values indicate greater average extinction risk. Together, panels highlight spatial variation in both the geographic expression of the CIP and the conservation severity of the native species involved.

The geographic structure of the CIP species was strongly asymmetric across regions (Fig. 4). When expressed as the ratio of non-native to native occurrences, all regions showed positive values (range: 1.54–5.64), indicating that each region functioned predominantly as a recipient rather than a donor within the CIP subset. This effect was strongest in Northwestern Europe (ratio = 5.64), Sub-Saharan Africa (5.13), and Oceania (3.22), whereas the lowest values were recorded in East-Southeast Asia (1.57), Eastern Europe (1.69), and North America (1.70) (Fig. 4A). In parallel, the mean threat score of native CIP species pools varied markedly among regions, ranging from 1.23 in Sub-Saharan Africa to 2.59 in South Asia. Higher average threat levels were concentrated in South Asia (2.59), West-Central Asia (2.50), and North America (2.50), whereas lower mean threat scores characterized Sub-Saharan Africa (1.23), Oceania (1.81), and Southern Europe (1.90) (Fig. 4B). Notably, the relationship between recipient bias and native threat severity was non-significant (Spearman r = −0.417, *p* = 0.178), indicating that the regions receiving the most CIP species are not necessarily those with the most threatened native CIP pools.

**Fig. 5:**
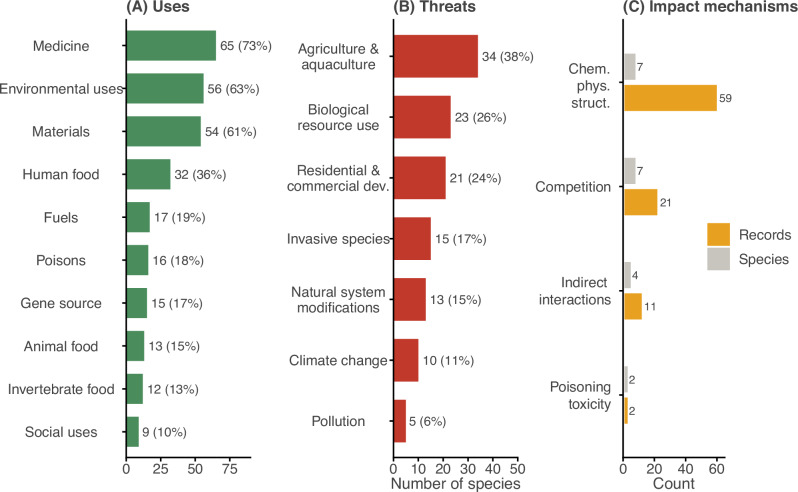
Human uses, threats, and ecological impacts of plant species exhibiting the Conservation–Invasion Paradox (CIP). **A** Number of CIP species recorded under each use category in the World Checklist of Useful Plant Species (WCUP 2020; *n* = 72 of 89 CIP species). **B** Number of CIP species facing each first-level IUCN threat category (IUCN Red List; *n* = 50 of 89 CIP species). **C** Number of species and impact records per impact mechanism documented in the Global Impacts Dataset of Invasive Alien Species (GIDIAS; *n* = 9 of 89 CIP species). Percentages in panels A and B are calculated relative to the total of 89 CIP species.

Information about uses, threats, and impacts of the 89 CIP species differed markedly among the three dimensions considered (Fig. 5). Data on human uses were available for 72 species (80.9%), encompassing ten distinct categories dominated by medicine (65 species), environmental uses (56), and materials (54), followed by human food (32) and fuels (17), whereas all remaining categories were less frequent (Fig. 5A). Threat information was documented for 50 species (56.2%) across seven first-level IUCN threat categories. The most common threats were agriculture and aquaculture (34 species), biological resource use (23), and residential and commercial development (21), followed by invasive and other problematic species (15), natural system modifications (13), climate change and severe weather (10), and pollution (5) (Fig. 5B). No statistically significant association was detected between use and threat categories among the 44 species with data available for both dimensions (permutation test: χ² = 24.37, *p* = 0.999; all pairwise Fisher’s exact tests non-significant after Benjamini–Hochberg correction). Information on ecological impacts was available for only 9 species (10.1%). The impact mechanisms documented included chemical, physical, or structural modifications of ecosystems and competition (7 species each), indirect effects mediated through interactions with other species (4 species), and poisoning or toxicity (2 species) (Fig. 5C).

## Discussion

This study provides the first systematic global assessment of the Conservation–Invasion Paradox (CIP) in vascular plants by identifying 89 species that simultaneously meet criteria for invasion outside their native ranges and conservation concern within them. While the previous work on the CIP has been mostly restricted to vertebrates,conceptual orcase-based analyses^12–15,17^, our results demonstrate that this paradox is also present in plants and detectable through reproducible cross-database criteria. Critically, the 89 CIP species identified are not merely co-occurrences of two rare conditions: threatened species are approximately 10 times less likely to naturalize and 17 times less likely to become invasive than non-threatened species. Importantly, the number of species identified here should be interpreted as a conservative minimum, constrained by data availability, taxonomic coverage, and the current structure of invasion and conservation databases. Evidence from taxa not captured by our study methods further supports this interpretation. For example, the coniferous *Juniperus communis*, which was not included in our analysis, has been reported as an emerging invader in southern South America^29^, while simultaneously experiencing demographic decline and genetic erosion across large portions of its native European range^30,31^, thus fulfilling the defining conditions of the CIP. More broadly, the 460 naturalized threatened plants identified in this study represent a pool of potential CIP species whose status warrants more investigation. Also, CIP species detected in this study are mainly distributed in intermediate conservation status categories, with a marked concentration toward lower-risk classes. Similar patterns have been documented in vertebrates, with invasive species showing a decoupling between expansion and persistence^15,17^. This is because the conservation status fundamentally reflects pressures operating in native ranges, while worldwide demographic processes for the species follow independent trajectories^12,13^.

When comparing the geographical origin of plants exhibiting CIP with the patterns reported for mammals (the only taxonomic group for which the geographical dimension of CIP has been explicitly evaluated^15^), it can be observed that CIP mammals’ native distributions are concentrated in Asia and Australia. In plants, native origins are mainly concentrated in the Americas and Oceania, with particularly high contributions from North America (*n* = 30) and East-Southeast Asia (28), while Northwestern Europe contributes comparatively few native CIP species (14). East-Southeast Asia and North America thus constitute regions of similar patterns between plants and mammals CIP, while the prominence of the Neotropics and Central America is specific to plants. This geographical structure is not surprising because it closely matches the global patterns reported for threatened vascular plants, which identify the Neotropics, Australasia and Indomalaya as regions with a high proportion of species^32,33^. More specifically, the concentration of CIP native origins in these regions is better explained by the life history and range characteristics typical of plants in high-diversity areas (including narrow endemic distributions, high habitat specialization, and restricted range sizes) which are precisely the traits that drive IUCN threat assessments under criteria based on area of occupancy and extent of occurrence^32,33^. In contrast, while global analyses of plant naturalization identify Europe as one of the regions over-represented as donors of non-native and invasive plants on a global scale^10,34,35^, this pattern is not reflected in the native origins of the CIP species detected in our study. Indeed, Northwestern Europe shows the highest recipient bias of all regions while contributing relatively few native CIP species. This suggests that the same region that exports invasive plants globally functions as a recipient within the CIP subset. This may show that CIP in plants is mainly associated with regions of high floristic vulnerability, characterized by narrow-ranged, specialized species with elevated extinction risk. The lack of relationship between recipient bias and native conservation status further supports this interpretation: the regions receiving the most non-native CIP species are not necessarily those where native CIP species face the greatest extinction risk, revealing a spatial decoupling between invasion success and conservation vulnerability.

Beyond its geographic expression, the same underlying processes linking human valuation and environmental pressure appear to be repeatedly expressed in CIP species. The patterns of human uses in CIP species indicate clear anthropogenic mediation with a predominance of materials, medicinal, and environmental uses. This is consistent with previously documented patterns^5,10,35^, where utilitarian value and deliberate translocation were identified as central drivers of the introduction and naturalization of plant species. At the same time, it weakens the idea of useful species being saved^36^. In turn, uses strongly associated with both increasing populations and high-risk categories reinforce the idea of a decoupling between human valuation, invasive population dynamics, and native persistence, in line with previous literature on CIP^12,13^. The dominant threats (agricultural activities, urbanization, fire, and climate change) are broadly consistent with the use profile of CIP species, suggesting that they face intensifying pressures in their native ranges precisely because of their socioeconomic value. The limited impact data available through GIDIAS, covering only 11 of the 89 CIP species, mirrors this pattern: the documented impact types, dominated by ecosystem modification, competition, and indirect interactions, are the mechanisms through which high-use species could reshape recipient landscapes. These results should nonetheless be interpreted with caution, as information about the type of threats was available for 53.9% of CIP species while data on impacts were available for just 10.1%. Both dimensions are characterized by substantial heterogeneity in documentation standards and data availability^37,38^. Thus, an expansion of systematic impact assessments such as EICAT^39^ is necessary to robustly assess the relationship between threat and impact categories in CIP species.

Given that invasive or naturalized populations outside the native range lack the complex biotic interactions, co-evolutionary histories, and ecological context that define their conservation value, the conservation of native populations must remain the primary objective. The genetic dimension of the CIP nonetheless opens an underexplored avenue for conservation planning. Importantly, this perspective should not be interpreted as promoting the maintenance or spread of invasive populations under any circumstances. Conservation efforts must remain firmly focused on preserving native populations; the potential use of introduced populations should be considered only opportunistically, drawing on those that persist despite management efforts and have not yet been fully eradicated, rather than encouraging their persistence or expansion. Introduced populations often undergo bottlenecks, multiple introductions, and rapid selection under distinct environmental conditions, leading to adaptive divergence from their source populations^40–42^. For example, *Pinus radiata* (one of the CIP species detected in this work) is native to a small area of coastal California and Mexico but globally cultivated for timber and considered invasive across multiple continents^43^. In its introduced range, rapid evolution has been documented in traits related to fire response and drought tolerance^44,45^, showing that invasive populations can retain or generate novel genetic diversity absent from native populations^44^. Meanwhile, native populations display high genetic singularity, signs of inbreeding, and demographic vulnerability associated with habitat fragmentation and erosion^46,47^. In this context, the systematic identification of CIP species can itself become a conservation tool: invasive populations may harbor genetic variants, locally adapted lineages, or allelic diversity no longer present in remnant native populations or ex situ collections. Crucially, the growing availability of genomic data in public repositories^48^ means that cross-referencing CIP lists with existing genetic databases could help conservation practitioners identify potential propagule sources with documented genetic provenance. Moreover, general population-level studies increasingly demonstrate that genetic diversity is declining globally, while targeted translocations and connectivity restoration can mitigate these losses^49^. However, any attempt to use introduced populations in native-range restoration must nonetheless be guided by rigorous assessment of adaptive divergence, outbreeding depression risk, and ecological compatibility, ensuring that the genetic contribution of invasive populations to conservation is both deliberate and ecologically sound^30^.

The CIP not only exposes the decoupling in the population dynamics of species between native and invaded ranges, but also highlights a disconnect between conservation and invasion management frameworks. While conservation policies prioritize the protection of threatened species in their native ranges, biosecurity strategies aim for their control or eradication elsewhere, creating conflicting priorities and a regulatory void for CIP species^20,50^. The IUCN Red List evaluates extinction risk without considering invasive potential, whereas invasion management frameworks rarely incorporate native-range conservation status^24^. International trade regulations offer limited resolution to this mismatch, as evidenced by the fact that only four CIP plant species identified in this study are currently listed under CITES appendices, despite widespread translocation driven by human use. This low representation suggests that most CIP taxa fall outside existing trade-based regulatory mechanisms, reinforcing the gap between conservation status, invasion risk, and global governance. Addressing these mismatches requires improved monitoring, coordination, and data integration between conservation and invasion frameworks, as recently emphasized in global calls for real-time tracking and harmonized assessment of biological invasions^21,51^. The CIP is closely related to the debate about assisted migration and ex-situ conservation^52^. In the case of plants, it remains questionable if introduced populations should be used for conservation as plant-soil and biotic interactions may be significantly altered, which can have negative long-term effects on the populations themselves, but also could cause extinctions in the recipient ecosystem^53^. Moving forward, effective management of CIP taxa requires a unified operational framework that bridges conservation and biosecurity. Such integration should include (i) explicit signaling of invasive potential within IUCN assessments, (ii) incorporation of native conservation status into trade and quarantine regulations, and (iii) sharing of demographic, genetic, and ecological data across ranges^13,20^. Control and eradication measures in invaded regions are usually guided by observed negative or uncertain impacts, whereas conservation actions in native ranges should focus on mitigating threats, restoring habitats, and maintaining genetic diversity. Due to this complexity, there are no simple solutions to reconcile both frameworks. In the case of CIP plants, this could mean that propagules from the non-native range could be collected and stored while management or control actions are implemented to reduce or eliminate those populations. This would allow their potential use as an opportunistic resource for restoration in the native range, although a solid understanding of their population genetics, as described above, is required to evaluate their suitability for such applications. Further, stronger coordination between conservation practitioners and researchers in native and invaded regions would be necessary.

The Conservation–Invasion Paradox illustrates that the categories of “threatened” and “invasive” species are not always opposites, but context-dependent expressions of the same anthropogenic processes operating across space. That threatened species are statistically far less likely to naturalize or invade than non-threatened ones makes the 89 species identified here genuinely paradoxical as empirically improbable outcomes of a global pattern in which conservation concern and invasion success are negatively associated. The same human-mediated pathways that facilitate population decline within native ranges frequently underpin population expansion elsewhere, exposing the limits of compartmentalized conservation and invasion frameworks. The genetic dimension explored in the previous paragraph adds a further layer to this duality: the introduced populations of CIP species may carry genetic conservation values that current frameworks are not designed to detect or incorporate into conservation planning. Recognizing this duality allows conservation science to move beyond fragmented approaches and to address extinction risk and biological invasions as interconnected outcomes of shared ecological, evolutionary, and socio-economic drivers. In this sense, the CIP does not represent an exception within conservation theory, but a predictable consequence of globalized species translocation acting upon uneven landscapes of vulnerability, which becomes a prerequisite for safeguarding long-term biodiversity persistence.

## Methods

### Data sources and integration

Because no single database captures the full diversity of invasive or threatened plant species globally (each source has different taxonomic and geographic biases) we built a multi-database framework integrating the most widely used non-native flora and conservation assessment databases to maximize species coverage. We integrated three global non-native flora databases (Laginhas & Bradley^23^; GRIIS^24^ and GloNAF 2.0^25^) with three conservation assessment databases (IUCN Red List^26^; BGCI ThreatSearch^27^ and NatureServe^28^) (Supplementary Table 1). Laginhas & Bradley (2022) compiled a consistently derived list of invasive plants by reviewing peer-reviewed publications from 1959 to 2020, applying a broad operational definition of invasiveness that encompasses spread, dominance, and impact criteria (Supplementary Table 1). GIDIAS^54^ was incorporated as an independent impact-based validation layer. GRIIS provided country-level checklists of introduced and invasive species, while GloNAF 2.0 documents naturalized non-native floras globally. Throughout this study, we retained the operational definition of each source database (Supplementary Table 1). For conservation status, the IUCN Red List v2024-2 provided global assessments for vascular plants. Due to known data gaps and biases in the IUCN Red List for plants^33^, we utilized the BGCI ThreatSearch aggregated global, regional, and national assessments from multiple sources; NatureServe provided assessments for North American vascular plants. All databases were downloaded in April 2026. Prior to integration, all databases were filtered to retain only species-level entries with a fully resolved binomial name, records identified only to genus level were excluded as they cannot be unambiguously assigned to a species. Hybrid taxa (indicated by the multiplication sign or “×” in the name) and intraspecific entries (varieties, subspecies, and forms) were removed as their conservation and invasion status is not consistently assessed across databases and to ensure that all comparisons were made at the species level. Further, duplicate records within each database — defined as multiple entries sharing the same accepted species name after taxonomic standardization — were collapsed to a single record per species. Taxonomic standardization was performed by matching all names against the GBIF Backbone Taxonomy using exact and fuzzy matching. For each database, the original species name was queried against the GBIF species API to retrieve the accepted name, taxonomic status, and confidence score of the match, retaining only names resolved to accepted species with a confidence score above 90, while synonyms were mapped to their accepted equivalents before joining. Cross-database matching was then performed on the standardized accepted names using exact string matching on genus and specific epithet in lowercase, excluding authorship. CITES appendix^55^ data were matched using the same procedure. The connection across databases was made at the species level, not at the country or sub-national level; country-level information from individual databases was used only for native and introduced range reconstruction, as described in the “Native and non-native range reconstruction” section of the Methods (see below). The resulting unified dataset comprised 124,414 species with documented conservation status, of which 13,696 had records as non-native species in at least one invasion database.

### Definition and identification of CIP species

We defined the Conservation–Invasion Paradox (CIP) as the simultaneous occurrence of invasive status outside the native range and conservation concern within the native range. CIP species were therefore classified as invasive in at least one non-native flora database and simultaneously listed under any category of conservation concern in at least one conservation database. Species were assigned to one of three risk levels: i) at risk: comprising NT, VU, EN, CR, EW (Table 1); ii) not at risk: corresponding to Least Concern; and iii) data deficient: which was excluded from all comparative analyses. Although EW (extinct in the wild) is not typically treated as a threatened category, it was included due to its ecological relevance. Conservation status was assigned following a hierarchical priority: IUCN Red List > NatureServe > BGCI ThreatSearch. To enable quantitative comparisons across these heterogeneous sources, we constructed a unified ordinal threat score: NT = 1, VU = 2, EN = 3, CR = 4, Extinct = 5 (Table 1), which was used to calculate mean threat scores and compare conservation severity across invasion stages and geographic regions. To contextualize and test the CIP within the broader invasion process, we additionally tracked species at earlier stages of establishment: naturalized (with self-sustaining populations outside the native range) and introduced (recorded outside the native range but not yet established), which together with invasive species comprised the full set of 610 threatened non-native plants identified in this study.

**Table 1:** Unified conservation status categories used in this study, their ordinal threat score, and the corresponding original categories from each source database

*Unified category*	*Threat score*	*IUCN Red List*	*BGCI*	*NatureServe*
Near Threatened (NT)	1	Near Threatened	*–*	G3(Vulnerable)
Vulnerable (VU)	2	Vulnerable	*–*	*–*
Endangered (EN)	3	Endangered	Threatened; Possibly Threatened	G1 (Critically Imperiled); G2 (Imperiled)
Critically Endangered (CR)	4	Critically Endangered	*–*	*–*
Extinct in the Wild (EW)	5	Extinct; Extinct in the Wild	Extinct	GX (Presumed Extinct); GH (Possibly Extinct)
Not at risk	*–*	Least Concern	Not Threatened	G4 (Apparently Secure); G5 (Secure)
Excluded	*–*	Data Deficient	Data Deficient	GU (Unrankable); GNR (Unranked); GNA (Not Applicable)

### Native and non-native range reconstruction

Native range data for each CIP species were obtained by cross-referencing records from IUCN Red List country distributions^26^, Laginhas & Bradley^23^ and Plants of the World Online^56^. Non-native ranges were reconstructed from GRIIS country-level checklists^24^, GloNAF 2.0^25^ regional records and Plants of the World Online^56^. Country-level data were aggregated to subcontinental and continental regions following standard geographic subdivisions. For each subcontinental region, we calculated the ratio of non-native to native CIP occurrences as a measure of recipient versus donor imbalance, and the mean threat score of native CIP species as a measure of conservation severity.

### Uses, threats, and impacts

Information on human uses was obtained from the World Checklist of Useful Plant Species^57^. Uses were obtained for 72 of the 89 CIP species, with the remaining 17 having no documented uses in this source. Threat information was extracted from IUCN Red List as^26^ available for 50 of the 89 CIP species; threat narratives were mapped to seven IUCN first-level threat categories following the IUCN Threats Classification Scheme^26,58^. Documented ecological impacts were obtained from the Global Impacts Dataset of Invasive Alien Species (GIDIAS)^54^, which compiles impact evidence from peer-reviewed literature following the EICAT framework^39^. Eleven CIP species were found in GIDIAS, of which eight had at least one impact mechanism documented; matching was performed using accepted GBIF names and synonyms to account for taxonomic reclassifications.

### Statistical analyses

To test whether threatened species were less likely to naturalize or become invasive than non-threatened species, we calculated odds ratios (OR) and 95% confidence intervals from 2 × 2 contingency tables comparing threatened versus non-threatened species across all assessed species. Significance was assessed using chi-square tests of independence. To evaluate whether the distribution of conservation categories shifted systematically along the invasion continuum, we computed mean threat scores and Spearman correlations between threat scores and species proportions across categories for four groups: all threatened species, those with records as naturalized species, and CIP species. A Cochran-Armitage trend test was used to assess whether naturalization and invasion rates decreased monotonically across the conservation status gradient from Least Concern to Critically Endangered. All analyses were conducted in Python (v3.12) using the pandas, scipy, numpy, and matplotlib libraries, and in R (v4.3.2) using the tidyverse, patchwork, countrycode, sf, and rnaturalearth packages. Geographic visualizations were produced by the authors using publicly available geographic boundaries accessed through the sf and rnaturalearth packages.

## Supplementary information

Supplementary information

## Data Availability

The raw datasets underpinning this study are based on publicly accessible sources cited in the Methods section and available through the IUCN database. The processed and cross-referenced dataset generated and analyzed during the current study is available as Supplementary Table 2.

## References

[1] Brym, Z. T., Lake, J. K., Allen, D. & Ostling, A. Plant functional traits suggest novel ecological strategy for an invasive shrub in an understorey woody plant community. *J. Appl. Ecol.* **48**, 1098–1106 (2011).

[2] Pyšek, P. et al. Disentangling the role of environmental and human pressures on biological invasions across Europe. *Proc. Natl. Acad. Sci.* **107**, 12157–12162 (2010). 20534543 10.1073/pnas.1002314107 PMC2901442

[3] Pyšek, P. et al. Naturalization of central European plants in North America: species traits, habitats, propagule pressure, residence time. *Ecology* **96**, 762–774 (2015). 26236872 10.1890/14-1005.1

[4] Catford, J. A., Jansson, R. & Nilsson, C. Reducing redundancy in invasion ecology by integrating hypotheses into a single theoretical framework. *Divers. Distrib.* **15**, 22–40 (2009).

[5] Hulme, P. E. Trade transport and trouble: managing invasive species pathways in an era of globalization. *J. Appl. Ecol.* **46**, 10–18 (2009).

[6] van Kleunen, M., Dawson, W. & Maurel, N. Characteristics of successful alien plants. *Mol. Ecol.* **24**, 1954–1968 (2015). 25421056 10.1111/mec.13013

[7] Blackburn, T. M., Cassey, P. & Duncan, R. P. Colonization pressure: a second null model for invasion biology. *Biol. Invasions* **22**, 1221–1233 (2020).

[8] Cassey, P., Delean, S., Lockwood, J. L., Sadowski, J. S. & Blackburn, T. M. Dissecting the null model for biological invasions: A meta-analysis of the propagule pressure effect. *PLOS Biol.* **16**, e2005987 (2018). 29684017 10.1371/journal.pbio.2005987 PMC5933808

[9] Colautti, R. I., Grigorovich, I. A. & MacIsaac, H. J. Propagule Pressure: A Null Model for Biological Invasions. *Biol. Invasions* **8**, 1023–1037 (2006).

[10] van Kleunen, M. et al. Economic use of plants is key to their naturalization success. *Nat. Commun.* **11**, 3201 (2020). 32581263 10.1038/s41467-020-16982-3 PMC7314777

[11] Kull, C. A., Tassin, J. & Rangan, H. Multifunctional, Scrubby, and Invasive Forests? *Mt. Res. Dev.* **27**, 224–231 (2007).

[12] Marchetti, M. P. & Engstrom, T. The conservation paradox of endangered and invasive species. *Conserv. Biol.* **30**, 434–437 (2016). 26954433 10.1111/cobi.12642

[13] Hong, Y., Yuan, Z. & Liu, X. Global drivers of the conservation–invasion paradox. *Conserv. Biol.* **39**, e14290 (2025). 38708868 10.1111/cobi.14290 PMC12451490

[14] Koster, S. et al. The conservation paradox of an introduced population of a threatened species: spadefoot toads in the coastal dunes of the Netherlands. *Amphib.-Reptil.* **44**, 11–18 (2022).

[15] Tedeschi, L. et al. Threatened Mammals With Alien Populations: Distribution, Causes, and Conservation. *Conserv. Lett.* **18**, e13069 (2025).

[16] Zhao, W. et al. Differences in characteristics between naturalized threatened plants and other threatened plants. *Conserv. Biol.* **40**, e70201 (2025). 41455029 10.1111/cobi.70201

[17] Rocha, C. F. D. & Bergallo, H. G. When invasive exotic populations are threatened with extinction. *Biodivers. Conserv.* **21**, 3729–3730 (2012).

[18] Bradshaw, C. J. A., Isagi, Y., Kaneko, S., Bowman, D. M. J. S. & Brook, B. W. Conservation Value of Non-Native Banteng in Northern Australia. *Conserv. Biol.* **20**, 1306–1311 (2006). 16922247 10.1111/j.1523-1739.2006.00428.x

[19] Lees, A. C. & Bell, D. J. A conservation paradox for the 21st century: the European wild rabbit Oryctolagus cuniculus, an invasive alien and an endangered native species. *Mammal Rev.* **38**, 304–320 (2008).

[20] Courchamp, F. et al. Invasion biology: specific problems and possible solutions. *Trends Ecol. Evol.* **32**, 13–22 (2017). 27889080 10.1016/j.tree.2016.11.001

[21] Guareschi, S. et al. Framing challenges and polarized issues in invasion science: toward an interdisciplinary agenda. *BioScience* **74**, 825–839 (2024). 39713562 10.1093/biosci/biae084 PMC11660934

[22] Gant, J. R., Mair, L. & McGowan, P. J. K. Fragmented evidence for the contribution of ex situ management to species conservation indicates the need for better reporting. *Oryx* **55**, 573–580 (2021).

[23] Laginhas, B. B. & Bradley, B. A. Global plant invaders: a compendium of invasive plant taxa documented by the peer-reviewed literature. *Ecology* **103**, e03569 (2022). 34699067 10.1002/ecy.3569

[24] Pagad, S., Genovesi, P., Carnevali, L., Schigel, D. & McGeoch, M. A. Introducing the Global Register of Introduced and Invasive Species. *Sci. Data* **5**, 170202 (2018). 29360103 10.1038/sdata.2017.202 PMC5779068

[25] Davis, A. J. S. et al. The updated Global Naturalized Alien Flora (GloNAF 2.0) database. *Ecology* **106**, e70245 (2025). 41195458 10.1002/ecy.70245

[26] IUCN - International Union for Conservation of Nature. *The IUCN Red List of Threatened Species - Data V2024-2*. https://www.iucnredlist.org (2024).

[27] BGCI. ThreatSearch online database. Botanic Gardens Conservation International (2024).

[28] NatureServe. NatureServe Explorer. NatureServe (2024).

[29] Franzese, J. & Ripa, R. R. Common juniper, an overlooked conifer with high invasion potential in protected areas of Patagonia. *Sci. Rep.* **13**, 9818 (2023). 37330618 10.1038/s41598-023-37023-1 PMC10276858

[30] Jacquemart, A.-L., Buyens, C., Delescaille, L.-M. & Van Rossum, F. Using genetic evaluation to guide conservation of remnant Juniperus communis (Cupressaceae) populations. *Plant Biol.* **23**, 193–204 (2021). 32991026 10.1111/plb.13188

[31] Verheyen, K. et al. Juniperus communis: victim of the combined action of climate warming and nitrogen deposition? *Plant Biol.* **11**, 49–59 (2009). 19778368 10.1111/j.1438-8677.2009.00214.x

[32] Bachman, S. P., Brown, M. J. M., Leão, T. C. C., Nic Lughadha, E. & Walker, B. E. Extinction risk predictions for the world’s flowering plants to support their conservation. *New Phytol.* **242**, 797–808 (2024). 38437880 10.1111/nph.19592

[33] Brummitt, N. A. et al. Green Plants in the Red: A Baseline Global Assessment for the IUCN Sampled Red List Index for Plants. *PLOS ONE* **10**, e0135152 (2015). 26252495 10.1371/journal.pone.0135152 PMC4529080

[34] Pouteau, R. et al. Climate and socio-economic factors explain differences between observed and expected naturalization patterns of European plants around the world. *Glob. Ecol. Biogeogr.* **30**, 1514–1531 (2021).

[35] van Kleunen, M. et al. Global exchange and accumulation of non-native plants. *Nature* **525**, 100–103 (2015). 26287466 10.1038/nature14910

[36] Mace, G. M. Whose conservation? *Science* **345**, 1558–1560 (2014). 25258063 10.1126/science.1254704

[37] Bachman, S. P. et al. Progress, challenges and opportunities for Red Listing. *Biol. Conserv.* **234**, 45–55 (2019).

[38] Bland, L. M. et al. Toward reassessing data-deficient species. *Conserv. Biol.* **31**, 531–539 (2017). 27696559 10.1111/cobi.12850

[39] Hawkins, C. L. et al. Framework and guidelines for implementing the proposed IUCN Environmental Impact Classification for Alien Taxa (EICAT). *Divers. Distrib.* **21**, 1360–1363 (2015).

[40] Dlugosch, K. M. & Parker, I. M. Founding events in species invasions: genetic variation, adaptive evolution, and the role of multiple introductions. *Mol. Ecol.* **17**, 431–449 (2008). 17908213 10.1111/j.1365-294X.2007.03538.x

[41] Estoup, A. et al. Is There a Genetic Paradox of Biological Invasion? *Annu. Rev. Ecol. Evol. Syst.* **47**, 51–72 (2016).

[42] Rius, M. & Darling, J. A. How important is intraspecific genetic admixture to the success of colonising populations? *Trends Ecol. Evol.* **29**, 233–242 (2014). 24636862 10.1016/j.tree.2014.02.003

[43] Mead, D. J. *Sustainable Management of Pinus Radiata Plantations*. (2013).

[44] Ripa, R. R. et al. Signs of rapid evolution in an invasive forest species: Drivers of the incipient neutral, adaptive and phenotypic divergence. *For. Ecol. Manag.* **546**, 121370 (2023).

[45] Williams, M. C. & Wardle, G. M. Pinus radiata invasion in Australia: Identifying key knowledge gaps and research directions. *Austral Ecol.* **32**, 721–739 (2007).

[46] Rogers, D. L., Jesús Vargas Hernández, J., Matheson, A. C. & Guerra Santos, J. J. Conserving the Pines of Guadalupe and Cedros Islands, Mexico: An International Collaboration. In *Environmental Issues in Latin America and the Caribbean* (eds Romero, A. & West, S. E.) 31–54 (Springer Netherlands, Dordrecht, 2005). 10.1007/1-4020-3774-0_2.

[47] Rogers, D. L., Matheson, A. C., Vargas-Hernández, J. J. & Guerra-Santos, J. J. Genetic Conservation of Insular Populations of Monterey Pine (Pinus Radiata D. Don). *Biodivers. Conserv.* **15**, 779–798 (2006).

[48] Theissinger, K. et al. How genomics can help biodiversity conservation. *Trends Genet.* **39**, 545–559 (2023). 36801111 10.1016/j.tig.2023.01.005

[49] Shaw, R. E. et al. Global meta-analysis shows action is needed to halt genetic diversity loss. *Nature* **638**, 704–710 (2025). 39880948 10.1038/s41586-024-08458-x PMC11839457

[50] Simberloff, D. et al. Impacts of biological invasions: what’s what and the way forward. *Trends Ecol. Evol.* **28**, 58–66 (2013). 22889499 10.1016/j.tree.2012.07.013

[51] Latombe, G. et al. A vision for global monitoring of biological invasions. *Biol. Conserv.* **213**, 295–308 (2017).

[52] Seddon, P. J. et al. The risks of assisted colonization. *Conserv. Biol.* **23**, 788–789 (2009). 19627304 10.1111/j.1523-1739.2009.01200.x

[53] Bucharova, A. Assisted migration within species range ignores biotic interactions and lacks evidence. *Restor. Ecol.* **25**, 14–18 (2017).

[54] Bacher, S. et al. Global Impacts Dataset of Invasive Alien Species (GIDIAS). *Sci. Data* **12**, 832 (2025). 40399318 10.1038/s41597-025-05184-5 PMC12095621

[55] CITES - Convention on International Trade in Endangered Species. Appendices I, II and III. *Convention on International Trade in Endangered Species (CITES)* https://cites.org/eng/app/index.php (2024).

[56] POWO. Plants of the World Online. *Facilitated by the Royal Botanic Gardens*, *Kew. Published on the Internet*. https://powo.science.kew.org/ (2025).

[57] Diazgranados, M. et al. World Checklist of Useful Plant Species.

[58] IUCN - International Union for Conservation of Nature. *IUCN Red List Categories and Criteria, Version 3.1, Second Edition*. (IUCN, 2012).

