# Supplementary material for: The rarest invaders: systematic global evidence for the conservation-invasion paradox in plants
Source: NPJ Biodivers. 2026 Jun 19;5:21. doi: 10.1038/s44185-026-00142-9 (PMC13284172; doi:10.1038/s44185-026-00142-9)
Supplement: Supplementary file 1 — Supplementary information [file 44185_2026_142_MOESM1_ESM.pdf]

Supplementary

The rarest invaders: systematic global evidence for the conservation-invasion paradox in plants

Ripa, Ramiro R.; Klinger, Yves P.; Franzese, Jorgelina

Supplementary Table 1. Overview of the six databases integrated to identify conservation–invasion paradox (CIP) species.

Invasion databases were used to flag species as invasive or naturalized outside their native range; conservation databases were used to assess extinction risk within native ranges. For each database, the operational definition or main criterion used to classify species is provided.

Database	Type	Geographic scope	Operational definition / main criterion
Laginhas & Bradley	Invasion	Global	Systematically derived list of invasive plants from peer-reviewed literature (1959–2020); broad definition of invasiveness encompassing spread, dominance, and impact criteria.
GRIIS	Invasion	Global (country-level)	Species recorded as invasive if there is documented evidence of negative impact on biodiversity; follows the IUCN definition: an alien species established in natural or semi-natural ecosystems that is an agent of change and threatens native biological diversity.
GloNAF 2.	Invasion	Global (regional-level)	Curated compilation of naturalized non-native vascular plant inventories; a species is considered naturalized when it has established self-sustaining populations in the wild outside its native range; invasive status retained as recorded in original regional sources (following Richardson et al. 2000 and Blackurn et al. 2011).
IUCN Red List v2024-2	Conservation	Global	Global extinction risk assessments using standardized quantitative criteria (population size, range extent, decline rates); categories: NT, VU, EN, CR, EW, EX.
BGCI ThreatSearch	Conservation	Global	Aggregates >300,000 conservation assessments from global, regional, and national sources; assigns an interpreted threat category to enable cross-system comparison
NatureServe	Conservation	North America	Conservation status assessments using a rank-based system (G1–G5) reflecting rarity, threats, and population viability for North American vascular plants.

Supplementary Table 2. Plant species exhibiting the conservation–invasion paradox (CIP).

Species (n = 89) meeting CIP criteria: invasive in ≥1 global invasion database (Laginhas & Bradley 2022 GPI, GRIIS, GloNAF v1) and at-risk in ≥1 conservation database (IUCN Red List, NatureServe Explorer, BGCI ThreatSearch). Conservation status follows the harmonized classification (See Methods). GIDIAS impact: whether documented invasion impacts exist in GIDIAS. Dashes indicate no data available. Use categories follow the World Checklist of Useful Plant Species (WCUP 2020, Kew Gardens).

Species	Conservation status	Uses	Threats	GIDIAS impact	Invasion databases (n)	Conservation databases (n)
Bartlettina sordida	Near Threatened	—	Agricultural activities; Deforestation; Livestock; Fires	No	2	1
Barleria prionitis	Near Threatened	Medicine	—	No	2	1
Anemone coronaria	Near Threatened	Medicine	—	No	3	1
Austrocylindropuntia cylindrica	Near Threatened	Human food; Environmental uses	Agriculture; Urban expansion; Livestock; Habitat loss	No	2	2
Campanula poscharskyana	Near Threatened	Environmental uses	—	No	3	1
Vitis rupestris	Vulnerable	Materials; Medicine	—	No	2	2
Robinia viscosa	Vulnerable	—	—	No	2	2
Coffea arabica	Endangered	Animal food; Human food; Gene source; Invertebrate food; Materials; Social uses; Medicine; Environmental uses	Agriculture; Climate change; Collection; Habitat conversion; Deforestation; Urban development; Diseases; Pathogens; Pests; Forest loss; Urbanization	No	3	3
Citharexylum ellipticum	Vulnerable	Medicine; Environmental uses	Agriculture; Land use change; Urban development; Pesticides; Pests; Urbanization	No	2	2
Cedrela odorata	Vulnerable	—	Deforestation; Logging; Habitat loss; Overharvesting	Yes	3	2
Brugmansia suaveolens	Extinct	Materials; Medicine; Environmental uses	Collection	No	3	3
Cinnamomum verum	Vulnerable	Human food; Fuel; Materials; Medicine; Environmental uses	Agricultural activities; Habitat conversion; Deforestation; Urbanization	No	3	3
Chromolaena bigelovii	Endangered	Medicine	—	No	1	2
Chasmanthe floribunda	Near Threatened	Medicine	—	No	3	1
Chamaecyparis lawsoniana	Near Threatened	Materials; Environmental uses; Medicine	Trade; Logging; Pathogens	No	2	3
Cotoneaster microphyllus	threatened	Materials; Environmental uses; Medicine	—	No	2	1

Cotoneaster glaucophyllus	Near Threatened	Environmental uses	—	No	2	1
Cupressus macrocarpa	Vulnerable	—	Climate change; Urban development; Fires; Urbanization	Yes	3	2
Gossypium hirsutum	Vulnerable	Gene source	Land use change; Habitat degradation; Urban development; Habitat loss	No	3	3
Gomphocarpus fruticosus	Near Threatened	Animal food; Human food; Gene source; Materials; Medicine; Environmental uses	—	No	3	1
Cyanthillium cinereum	Near Threatened	Poison; Materials; Medicine	—	No	3	1
Dianthus giganteus	Near Threatened	Medicine	—	No	2	1
Dioscorea oppositifolia	Near Threatened	Materials; Medicine; Human food	—	No	2	1
Cryptomeria japonica	Near Threatened	Materials; Environmental uses; Medicine	Climate change	No	3	3
Dypsis lutescens	Near Threatened	Gene source; Human food; Environmental uses	Agricultural activities; Habitat loss; Overharvesting	No	3	2
Fraxinus pennsylvanica	Critically Endangered	Materials; Environmental uses; Medicine	Climate change	Yes	2	3
Fraxinus americana	Critically Endangered	Materials; Medicine; Environmental uses	Climate change	Yes	2	3
Erigeron canadensis	Near Threatened	Human food; Fuel; Materials; Medicine; Poison	—	Yes	3	1
Eucalyptus cladocalyx	Vulnerable	Materials; Environmental uses; Poison	Agricultural activities	No	3	3
Eucalyptus cinerea	Near Threatened	Materials; Environmental uses; Medicine	Agricultural activities	No	3	3
Eucalyptus camaldulensis	Near Threatened	Animal food; Human food; Fuel; Invertebrate food; Materials; Medicine; Environmental uses	Agricultural activities	Yes	3	3
Eucalyptus botryoides	Near Threatened	Invertebrate food; Materials; Environmental uses; Medicine	Agricultural activities; Urbanization	No	3	3
Eucalyptus benthamii	Endangered	Materials	Agricultural activities; Urban development; Urbanization	No	1	2
Aristolochia bodamae	threatened	Medicine	—	No	1	1

Ferraria crispa	Near Threatened	Social uses; Human food; Medicine	—	No	2	1
Eucalyptus robusta	Near Threatened	Materials	Agricultural activities; Urbanization	No	3	3
Eucalyptus megacornuta	Vulnerable	Materials	The association between potential threats and population declines requires further assessment.	No	2	2
Eucalyptus gunnii	Endangered	Materials; Environmental uses	Climate change; Grazing	No	2	2
Eucalyptus grandis	Near Threatened	Human food; Fuel; Materials; Invertebrate food; Medicine; Environmental uses	Agricultural activities; Urbanization	Yes	3	3
Eucalyptus gomphocephala	Vulnerable	Fuel; Materials; Invertebrate food; Medicine; Environmental uses	Agricultural activities; Urban development; Urbanization	No	3	3
Eucalyptus conferruminata	Near Threatened	Materials	Urban development; Urbanization	No	2	2
Eucalyptus viminalis	Near Threatened	Materials	Agricultural activities	No	3	2
Eucalyptus urophylla	Endangered	Materials	Agricultural activities; Habitat conversion	No	2	2
Ulmus americana	Endangered	Materials; Environmental uses; Medicine	Climate change; Diseases; Invasive species	No	2	3
Juniperus bermudiana	Critically Endangered	Materials; Environmental uses	Invasive species; Logging; Urbanization	No	3	2
Jasione crispa	threatened	Medicine; Environmental uses	—	No	1	1
Jacaranda mimosifolia	Vulnerable	Materials	Agricultural activities; Logging	Yes	3	3
Juglans cinerea	Endangered	Gene source	Climate change; Diseases; Pathogens; Pests; Habitat loss	No	2	3
Kunzea ericoides	Near Threatened	Materials; Medicine	Agriculture; Diseases; Invasive species; Fires; Urbanization	No	2	2
Lathyrus odoratus	Critically Endangered	Poison; Human food; Gene source; Materials; Medicine; Environmental uses	Collection	No	3	3
Kalanchoe daigremontiana	Endangered	Environmental uses; Poison	Agricultural activities; Habitat degradation; Fires; Grazing	Yes	3	2
Lupinus arboreus	Vulnerable	Medicine	—	No	3	2
Linum hirsutum	threatened	Gene source	—	No	1	1
Mahonia aquifolium	Near Threatened	—	—	No	1	1
Mahonia repens	threatened	—	—	No	1	1

Metrosideros polymorpha	Vulnerable	Invertebrate food; Materials; Environmental uses; Medicine	Habitat degradation; Predation; Diseases; Invasive species	No	1	2
Nymphaea mexicana	Vulnerable	Human food; Medicine; Materials; Social uses; Gene source; Animal food; Environmental uses	—	No	2	2
Nicotiana mutabilis	threatened	Gene source	—	No	1	1
Murraya paniculata	Near Threatened	Human food; Gene source; Invertebrate food; Materials; Social uses; Medicine; Environmental uses	—	No	3	1
Penstemon oliganthus	Vulnerable	Environmental uses	—	No	1	2
Phytolacca rivinoides	threatened	Medicine; Human food	—	No	2	1
Pinus peuce	Near Threatened	Materials; Environmental uses	Diseases; Fires	No	2	2
Pinus muricata	Vulnerable	Fuel; Materials; Environmental uses	Fires; Urbanization	No	2	3
Pinus monticola	Near Threatened	Materials	Fires; Overharvesting	No	1	3
Piper umbellatum	threatened	Animal food	—	No	2	1
Prosopis tamarugo	threatened	Fuel; Materials; Environmental uses; Animal food	—	No	1	1
Primula maximowiczii	Near Threatened	Materials	—	No	1	1
Phytolacca acinosa	threatened	Human food; Medicine; Materials; Gene source; Poison; Animal food; Environmental uses	—	No	2	1
Potentilla matsumurae	Near Threatened	Medicine	—	No	1	1
Pterocarpus indicus	Endangered	Poison	Trade; Logging	No	2	2
Polygala paniculata	threatened	Materials; Medicine	—	No	3	1
Pterocarpus macrocarpus	Endangered	Medicine; Environmental uses	Agricultural activities; Habitat conversion; Deforestation; Logging; Fires; Grazing; Overharvesting; Urbanization	No	2	2
Reutealis trisperma	Near Threatened	Human food; Materials; Environmental uses; Medicine; Poison	Agricultural activities; Deforestation; Overharvesting; Urbanization	No	2	2

Rhododendron ponticum	Near Threatened	Environmental uses; Gene source	—	Yes	2	1
Pinus radiata	Endangered	Materials; Environmental uses; Medicine; Human food	Fires; Pathogens	Yes	3	3
Sesbania herbacea	Near Threatened	Materials; Environmental uses; Poison	—	No	2	1
Saxifraga jacquemontiana	threatened	Materials; Medicine; Environmental uses	—	No	1	1
Solanum chenopodioides	Near Threatened	Gene source	—	No	2	1
Solidago virgaurea	threatened	Medicine	—	No	1	1
Zingiber montanum	Near Threatened	Medicine	—	No	2	1
Sorbaria tomentosa	Extinct	—	—	No	2	1
Tectona grandis	Endangered	—	Agricultural activities; Climate change; Land use change; Trade; Logging; Fires; Grazing; Pests; Overharvesting; Urbanization	No	2	3
Camonea umbellata	Near Threatened	—	—	No	1	1
Coccothrinax barbadensis	Near Threatened	Materials; Environmental uses	—	No	2	1
Eucalyptus pulchella	Near Threatened	Materials	Agricultural activities; Urbanization	No	2	2
Myosotis azorica	Vulnerable	Animal food	Agricultural activities	No	2	3
Pyracantha koidzumii	Endangered	—	Trade	No	1	2
Santalum album	Vulnerable	Materials; Medicine	Diseases; Fires; Grazing; Overharvesting	No	2	2
Vanilla planifolia	Endangered	Human food; Gene source; Materials; Medicine; Environmental uses	Collection	No	2	2
